# Sparse Representation-Based Multi-Focus Image Fusion Method via Local Energy in Shearlet Domain

**DOI:** 10.3390/s23062888

**Published:** 2023-03-07

**Authors:** Liangliang Li, Ming Lv, Zhenhong Jia, Hongbing Ma

**Affiliations:** 1 Department of Electronic Engineering, Tsinghua University, Beijing 100084, China; 2 College of Information Science and Engineering, Xinjiang University, Urumqi 830046, China

**Keywords:** multi-focus image, image fusion, local energy, sparse representation, shearlet

## Abstract

Multi-focus image fusion plays an important role in the application of computer vision. In the process of image fusion, there may be blurring and information loss, so it is our goal to obtain high-definition and information-rich fusion images. In this paper, a novel multi-focus image fusion method via local energy and sparse representation in the shearlet domain is proposed. The source images are decomposed into low- and high-frequency sub-bands according to the shearlet transform. The low-frequency sub-bands are fused by sparse representation, and the high-frequency sub-bands are fused by local energy. The inverse shearlet transform is used to reconstruct the fused image. The Lytro dataset with 20 pairs of images is used to verify the proposed method, and 8 state-of-the-art fusion methods and 8 metrics are used for comparison. According to the experimental results, our method can generate good performance for multi-focus image fusion.

## 1. Introduction

Due to the limited depth of field of the optical lens, the imaging device sometimes cannot achieve clear focus imaging of all objects or areas in the same scene, resulting in defocus and blurring of the scene content outside the depth of field [1,2,3,4,5]. In order to solve the above problems, multi-focus image fusion technology provides an effective way to synthesize the complementary information contained in multiple partially focused images in the same scene, and then generate an all-in-focus fusion image, which is more suitable for human observation or computer processing, and has wide application value in digital photography, microscopic imaging, holographic imaging, integrated imaging, and other fields [6,7,8,9,10,11,12,13,14,15].

Now, many multi-focus image fusion methods have been proposed. Especially, the methods based on multi-scale transform, sparse representation, edge-preserving filtering, and deep learning have achieved remarkable results in image fusion [16]. The curvelet [17], surfacelet [18], contourlet [19,20], and shearlet transforms [21,22,23] are widely used in multi-scale transform fields. Vishwakarma et al. [24] introduced the multi-focus image fusion algorithm via curvelet transform and the Karhunen–Loève Transform (KLT), and this method can achieve fused images with less noise and improve the information interpretation capability of the fused images. Yang et al. [25] proposed the multi-focus image fusion method using a pulse-coupled neural network (PCNN) and sum-modified-Laplacian algorithms in the fast discrete curvelet transform domain. Zhang et al. [26] proposed a multi-focus image fusion technique using a compound pulse-coupled neural network in a surfacelet domain, with a local sum-modified-Laplacian algorithm used as the external stimulus of the compound PCNN, and the results show that this method can achieve a good performance for multi-focus image fusion. Li et al. [27] introduced multi-focus image fusion utilizing dynamic threshold neural P systems and a surfacelet transform, and the sum-modified- Laplacian algorithm and spatial frequency are regarded as the external inputs of dynamic threshold neural P systems for low- and high-frequency coefficients, respectively; consistent verification is used to obtain the final multi-focus fused image, and this method can solve the problem of artifacts. Xu et al. [28] introduced an image fusion utilizing an enhanced cross-visual cortex model based on artificial selection and an impulse-coupled neural network in a nonsubsampled contourlet transform domain. This method can achieve outstanding edge information, high contrast, and brightness. Das et al. [29] introduced a fuzzy-adaptive reduced pulse-coupled neural network for image fusion in a nonsubsampled contourlet transform domain, and this method can generate a fused image with higher contrast than other state-of-the-art image fusion algorithms. Li et al. [30] introduced the multi-focus image fusion framework using multi-scale transform decomposition, where the nonsubsampled contourlet transform is used to obtain the basic fused image, and the energy of gradient of difference images is used to refine the basic fused image by integrating the average filter and median filter. This method can generate a high-definition fused image. Peng et al. [31] proposed coupled neural P systems and a nonsubsampled contourlet transform for image fusion, and the quantitative and qualitative experimental results demonstrate the advantages of the fusion approach. Wang et al. [32] introduced the complex shearlet features-motivated generative adversarial network for multi-focus image fusion. Li et al. [33] proposed one multi-focus image fusion method via spatial frequency-motivated parameter-adaptive pulse-coupled neural network and an improved sum-modified- Laplacian in nonsubsampled shearlet transform domain, and visual inspection and objective evaluation verified the effectiveness of the fusion method. Amrita et al. [34] proposed an image fusion method using a water wave optimization (WWO) algorithm in a nonsubsampled shearlet transform domain, and this method can obtain good fusion results. Luo et al. [35] introduced multi-modal image fusion using a 3-D shearlet transform and T-S fuzzy reasoning, and this method can achieve good fusion results. Yin et al. [36] proposed the parameter-adaptive pulse-coupled neural network (PAPCNN)-based multi-modal image fusion method in a nonsubsampled shearlet transform domain, where the weighted local energy and weighted sum of eight- neighborhood-based modified Laplacian algorithms are used for fusing the low-frequency components, and the PAPCNN-based fusion model is used for fusing the high-frequency components; the results generate state-of-the-art performance, according to the visual perception and objective assessments.

The sparse representation-based methods have been widely used in image restoration and image fusion [37,38,39,40,41,42,43,44,45,46]. Wang et al. [47] proposed a joint patch clustering-based adaptive dictionary and sparse representation for multi-modality image fusion, where the Gaussian filter is used to separate the low- and high-components, the local energy-weighted strategy is used to fuse the low-frequency sub-bands, an over-complete adaptive learning dictionary is reconstructed by the joint patch clustering model, and a hybrid fusion rule depending on the similarity of the multi-norm of sparse representation coefficients is introduced to fuse the high-frequency sub-bands. This method has good robustness and wide application. Qin et al. [48] proposed an improved image fusion algorithm using a discrete wavelet transform and sparse representation, and this method can achieve higher contrast and more image details. Liu et al. [49] introduced an effective image fusion approach using convolutional sparse representation, and this method outperforms other image fusion algorithms in terms of visual and objective assessments. Liu et al. [50] introduced an adaptive sparse representation model for multi-focus image fusion and denoising, and this approach generates good performance, according to the visual quality and objective assessment.

Edge-preserving filtering has been widely used in image enhancement, image smoothing, image denoising, and image fusion. Especially in the field of image fusion, it has a very significant effect. Li et al. [51] introduced guided image filtering for image fusion. The base layer and detail layer are generated by guided image filtering decomposition, and the weighted average model is used as the fusion rule. This method is used in experiments on muti-spectral, multi-focus, multi-modal, and multi-exposure images for fusion, and it can obtain fast and effective fusion results. Zhang et al. [52] introduced local extreme map guided image filtering for image fusion, such as medical images, multi-focus images, infrared and visual images, and multi-exposure images, and this method can generate good performance.

Deep learning-based image fusion methods have been widely used in image processing. Zhang et al. [53] proposed an image fusion method using a convolutional neural network (IFCNN), and this method has good performance for multi-focus, infrared-visual, multi-modal medical and multi-exposure image fusion. Zhang et al. [54] introduced a fast unified image fusion network based on the proportional maintenance of gradient and intensity, and this method can generate good fusion results. Xu et al. [55] proposed the unified and unsupervised end-to-end image fusion network (U2Fusion), and this algorithm achieves better fusion effects compared to state-of-the-art fusion methods. Dong et al. [56] proposed a multi-branch multi-scale deep learning image fusion algorithm based on denseNet, and this method can achieve excellent results and keep more feature information of the source images in the fused image.

In order to generate a high-quality multi-focus fusion image, a novel image fusion framework based on sparse representation and local energy is proposed. The source images are separated into the low- and high-frequency sub-bands by shearlet transform, then the sparse representation model is used for fusing the low-frequency sub-bands, and the local energy-based fusion rule is used for fusing the high-frequency sub-bands. The inverse shearlet transform is applied to reconstruct the fused image. Experimental results show that the proposed multi-focus image fusion method can retain more source image information.

## 2. Related Works

### 2.1. Shearlet Transform

In dimension n=2, the shearlet transform (ST) for the signal f can be defined as follows [21]:

(1) $$SH_{\psi }\left(f\right)=\langle f,\psi_{a,s,t}\rangle$$

where $SH_{\psi }\left(\cdot \right)$ shows the shearlet transform. 〈⋅〉 depicts the inner product. The ST projects f onto the functions $\psi_{a,s,t}$ at scale a, orientation s, and location t.

The element $\psi_{a,s,t}$ is named shearlet, and it can be generated by:

(2) $$\psi_{a,s,t}\left(x\right)={\left|\det M_{a,s}\right|}^{-\frac{1}{2}}\psi \left(M_{a,s}^{-1}x-t\right)\;a\in R^{+},\;s\in R,\;t\in R^{2}$$

where the parameters $R^{+}$, R, and $R^{2}$ show the positive real numbers, real numbers, and 2-dimensional real vectors, respectively. $M_{a,s}$ can be computed by:

(3) $$M_{a,s}=\left(\begin{array}{l} a\sqrt{a}s\\ 0\sqrt{a} \end{array}\right)$$

where $M_{a,s}=S_{s}A_{a}$ consists of two matrixes: the shear transform matrix $S_{s}$ and the anisotropic dilation matrix $A_{a}$. The corresponding equations can be computed by:

(4) $$S_{s}=\left(\begin{array}{l} 1\;s\\ 0\;1 \end{array}\right)$$

(5) $$A_{a}=\left(\begin{array}{l} a\;\;\;\;0\\ 0\;\sqrt{a} \end{array}\right)$$

The inverse shearlet transform is computed by:

(6) $$f=\int_{R^{2}}\int_{-\infty }^{\infty }\int_{0}^{\infty }\langle f,\psi_{a,s,t}\rangle \psi_{a,s,t}\frac{da}{a^{3}}dsdt$$

### 2.2. Sparse Representation

Sparse representation can effectively extract the essential characteristics of signals and can be represented by a linear combination of non-zero atoms in a set of dictionaries [57]. We define the signal $x\in R^{n}$ and the over-complete dictionary $D\in R^{n\times m}\left(n<m\right)$. The purpose of sparse representation is to estimate the sparse vector $\alpha \in R^{m}$ with the fewest nonzero entries, such that x≈Dα. Suppose that M training patches of size $\sqrt{n}\times \sqrt{n}$ are rearranged to column vectors in the $R^{n}$ space, so the training database ${\left\{y_{i}\right\}}_{i=1}^{M}$ is constructed with each $y_{i}\in R^{n}$. The dictionary learning model can be depicted as follows:

(7) $$\underset{D,\;{\left\{\alpha_{i}\right\}}_{i=1}^{M}}{\min}\sum_{i=1}^{M}{\left\|\alpha_{i}\right\|}_{0}\;\;\;s.t.{\left\|y_{i}-D\alpha_{i}\right\|}_{2}<\varepsilon ,\;\;i\in \left\{1,\;.\;.\;.\;,M\right\}$$

where ε>0 shows an error tolerance, ${\left\{\alpha_{i}\right\}}_{i=1}^{M}$ shows the unknown sparse vectors corresponding to ${\left\{y_{i}\right\}}_{i=1}^{M}$, and $D\in R^{n\times m}$ is the unknown dictionary to be learned. Some effective models, such as MOD and K-SVD, have been introduced to deal with this question. More details can be seen in reference [57].

## 3. Proposed Fusion Method

**Figure 1:**
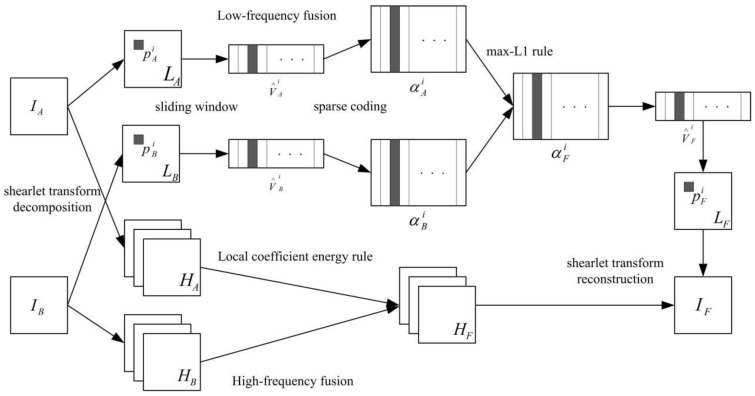
Diagram of the proposed fusion method.

The proposed image fusion algorithm mainly contains four phases: shearlet transform decomposition, low-frequency fusion, high-frequency fusion, and shearlet transform reconstructed. The schematic diagram of the proposed approach is described in Figure 1.

### 3.1. Shearlet Transform Decomposition

The shearlet transform decomposition performs on the two source images $\left\{I_{A},I_{B}\right\}$ to achieve the low-frequency components $\left\{L_{A},L_{B}\right\}$ and the high-frequency components $\left\{H_{A},H_{B}\right\}$.

### 3.2. Low-Frequency Fusion

In the low-frequency component, the main energy of the image is concentrated, and the subject of the image is in the low-frequency component. In this section, $L_{A}$ and $L_{B}$ are merged with the sparse representation fusion method. The sliding window method is utilized to divide $L_{A}$ and $L_{B}$ into image patches with the size $\sqrt{n}\times \sqrt{n}$ from upper left to lower right with the step length of s pixels. Assume that there are T patches depicted as ${\left\{p_{A}^{i}\right\}}_{i=1}^{T}$ and ${\left\{p_{B}^{i}\right\}}_{i=1}^{T}$ in $L_{A}$ and $L_{B}$, respectively.

For each position i, rearrange $\left\{p_{A}^{i},p_{B}^{i}\right\}$ into column vectors $\left\{v_{A}^{i},v_{B}^{i}\right\}$ and then normalize each vector’s mean value to zero to obtain $\left\{{\hat{V}}_{A}^{i},{\hat{V}}_{B}^{i}\right\}$ by the following equations [57]:

(8) $${\hat{V}}_{A}^{i}=V_{A}^{i}-{\bar{v}}_{A}^{i}\cdot 1$$

(9) $${\hat{V}}_{B}^{i}=V_{B}^{i}-{\bar{v}}_{B}^{i}\cdot 1$$

where 1 shows an all-one valued n×1 vector, and ${\hat{v}}_{A}^{i}$ and ${\hat{v}}_{B}^{i}$ are the mean values of all the elements in $V_{A}^{i}$ and $V_{B}^{i}$, respectively.

For the sparse coefficient vectors $\left\{\alpha_{A}^{i},\alpha_{B}^{i}\right\}$ of $\left\{{\hat{V}}_{A}^{i},{\hat{V}}_{B}^{i}\right\}$, we can compute them utilizing the orthogonal matching pursuit (OMP) technique with the following formulas:

(10) $$\alpha_{A}^{i}=\arg\underset{\alpha }{\min}{\left\|\alpha \right\|}_{0}\;s.t.\;{\left\|{\hat{V}}_{A}^{i}-D\alpha \right\|}_{2}<\varepsilon$$

(11) $$\alpha_{B}^{i}=\arg\underset{\alpha }{\min}{\left\|\alpha \right\|}_{0}\;s.t.\;{\left\|{\hat{V}}_{B}^{i}-D\alpha \right\|}_{2}<\varepsilon$$

where D denotes the learned dictionary that is trained by the *K*-singular value decomposition (*K*-SVD) method.

Then, $\alpha_{A}^{i}$ and $\alpha_{B}^{i}$ are merged with the “max-L1” rule to obtain the fused sparse vector:

(12) $$\alpha_{F}^{i}=\left\{ \begin{array}{l} \alpha_{A}^{i}\;\;if{\left\|\alpha_{A}^{i}\right\|}_{1}>{\left\|\alpha_{B}^{i}\right\|}_{1}\\ \alpha_{B}^{i}\;\;else \end{array}\right.$$

The fused results of $V_{A}^{i}$ and $V_{B}^{i}$ can be computed by the following:

(13) $$V_{F}^{i}=D\alpha_{F}^{i}+{\hat{v}}_{F}^{i}\cdot 1$$

where the merged mean value ${\bar{v}}_{F}^{i}$ can be calculated by the following:

(14) $${\bar{v}}_{F}^{i}=\left\{ \begin{array}{l} {\bar{v}}_{A}^{i}\;\;if\;\alpha_{F}^{i}=\alpha_{A}^{i}\\ {\bar{v}}_{B}^{i}\;\;else \end{array}\right.$$

The above process is iterated for all the source image patches in ${\left\{p_{A}^{i}\right\}}_{i=1}^{T}$ and ${\left\{p_{B}^{i}\right\}}_{i=1}^{T}$ to obtain all the fused vectors ${\left\{V_{F}^{i}\right\}}_{i=1}^{T}$. Let $L_{F}$ denote the low-pass fused result. For each $V_{F}^{i}$, reshape it into a patch $p_{F}^{i}$ and then plug $p_{F}^{i}$ into its original position in $L_{F}$. As patches are overlapped, each pixel’s value in $L_{F}$ is averaged over its accumulation times.

### 3.3. High-Frequency Fusion

The high-frequency components contain a great deal of detailed information, and the high-frequency components are fused using the local coefficient energy, which is described as follows [58]:

(15) $$E\left(\omega \right)=\sum_{m=1}^{M}\sum_{n=1}^{N}H{\left(m,n\right)}^{2}/\left(M\times N\right)$$

where H(m,n) represents the high-frequency coefficients at pixel (m,n), and ω is a local window with size M×N. Let $\omega_{A}\left(i,j\right)$ and $\omega_{B}\left(i,j\right)$ show the local windows centered at pixel (i,j) in $H_{A}$ and $H_{B}$, respectively. The high-frequency fused result $H_{F}$ is achieved by the following:

(16) $$H_{F}\left(i,j\right)=\left\{ \begin{array}{l} H_{A}\left(i,j\right)\;\;E\left(\omega_{A}\left(i,j\right)\right)>E\left(\omega_{B}\left(i,j\right)\right)\\ H_{B}\left(i,j\right)\;\;else \end{array}\right.$$

### 3.4. Shearlet Transform Reconstruction

The inverse shearlet transform is performed on $L_{F}$ and $H_{F}$ to reconstruct the final fused image $I_{F}$.

## 4. Experimental Results and Discussions

**Figure 2:**
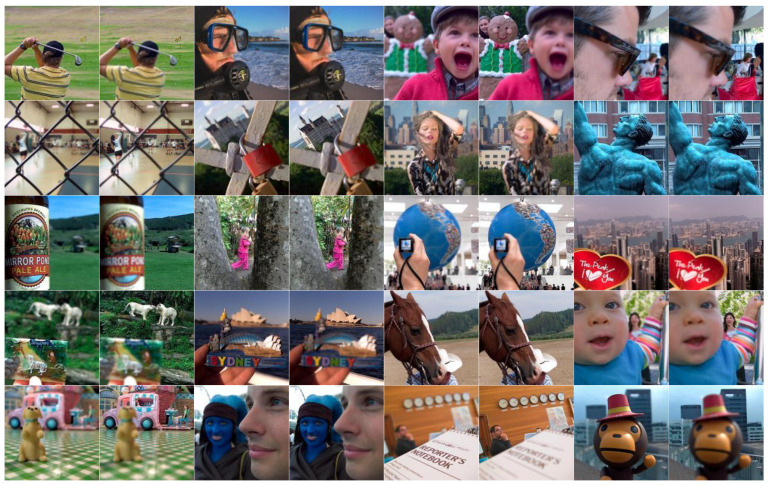
Lytro dataset.

**Figure 3:**
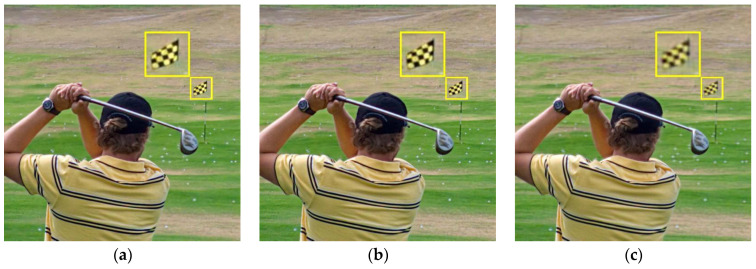

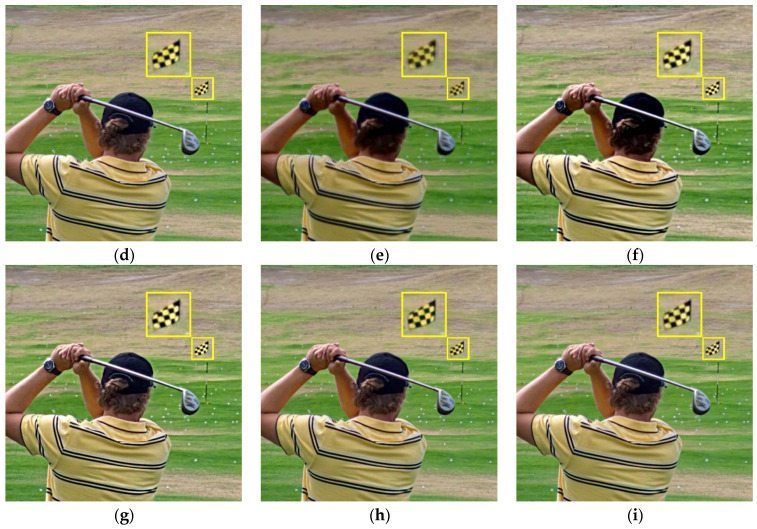
Fusion results on the first pair of images. (**a**) NSCT; (**b**) CVT; (**c**) NSST; (**d**) IFCNN; (**e**) PMGI; (**f**) U2Fusion; (**g**) LEGFF; (**h**) ZMFF; (**i**) Proposed.

**Figure 4:**
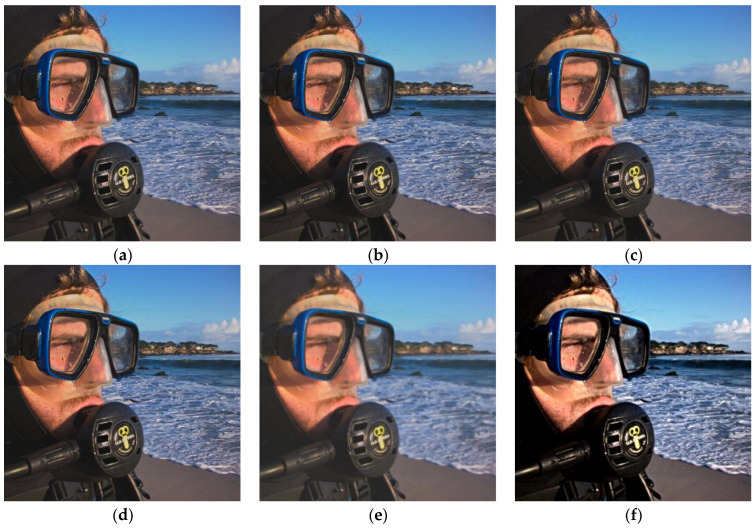

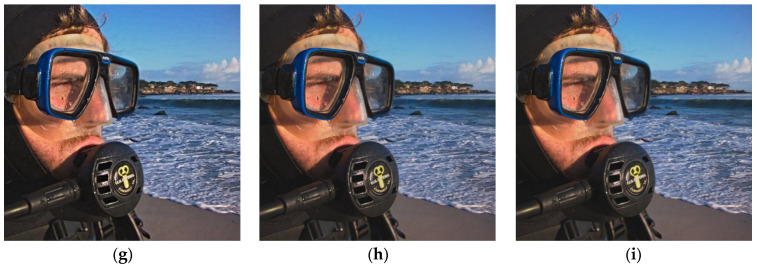
Fusion results on the second pair of images. (**a**) NSCT; (**b**) CVT; (**c**) NSST; (**d**) IFCNN; (**e**) PMGI; (**f**) U2Fusion; (**g**) LEGFF; (**h**) ZMFF; (**i**) Proposed.

**Figure 5:**
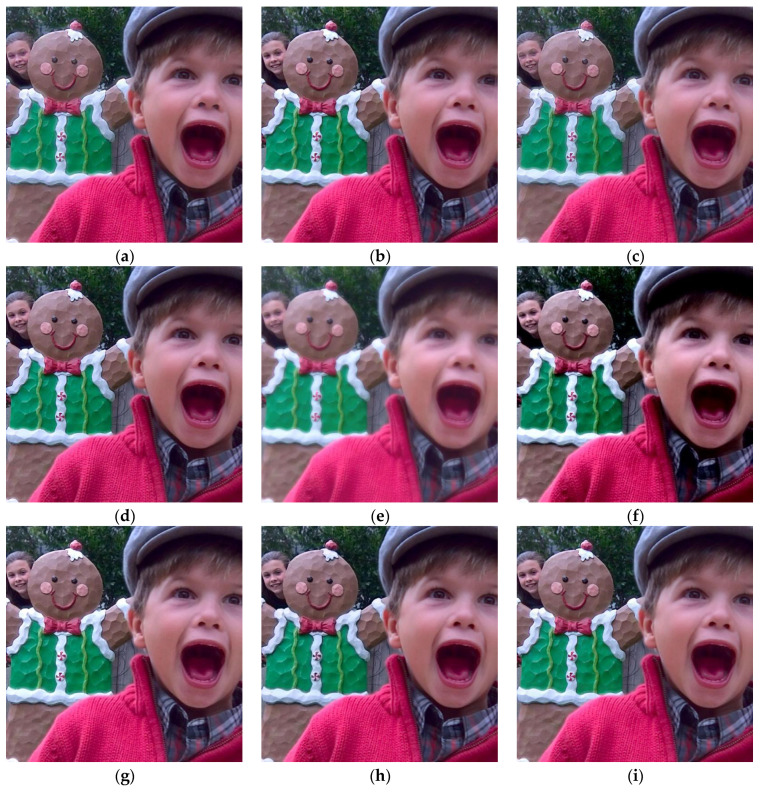
Fusion results on the third pair of images. (**a**) NSCT; (**b**) CVT; (**c**) NSST; (**d**) IFCNN; (**e**) PMGI; (**f**) U2Fusion; (**g**) LEGFF; (**h**) ZMFF; (**i**) Proposed.

**Figure 6:**
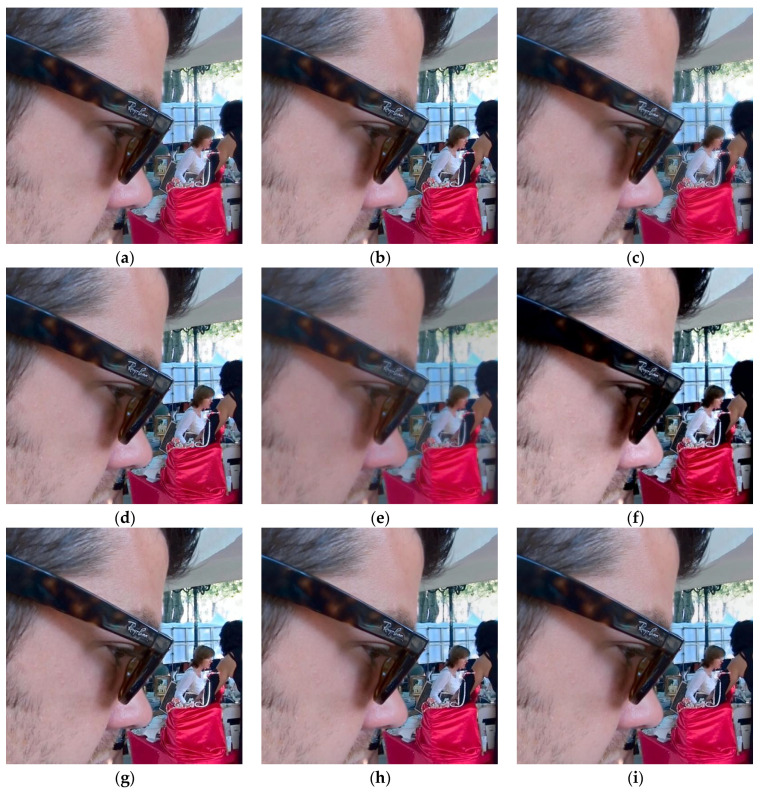
Fusion results on the fourth pair of images. (**a**) NSCT; (**b**) CVT; (**c**) NSST; (**d**) IFCNN; (**e**) PMGI; (**f**) U2Fusion; (**g**) LEGFF; (**h**) ZMFF; (**i**) Proposed.

**Figure 7:**
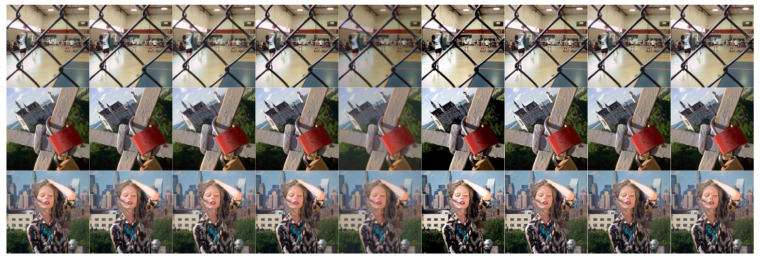

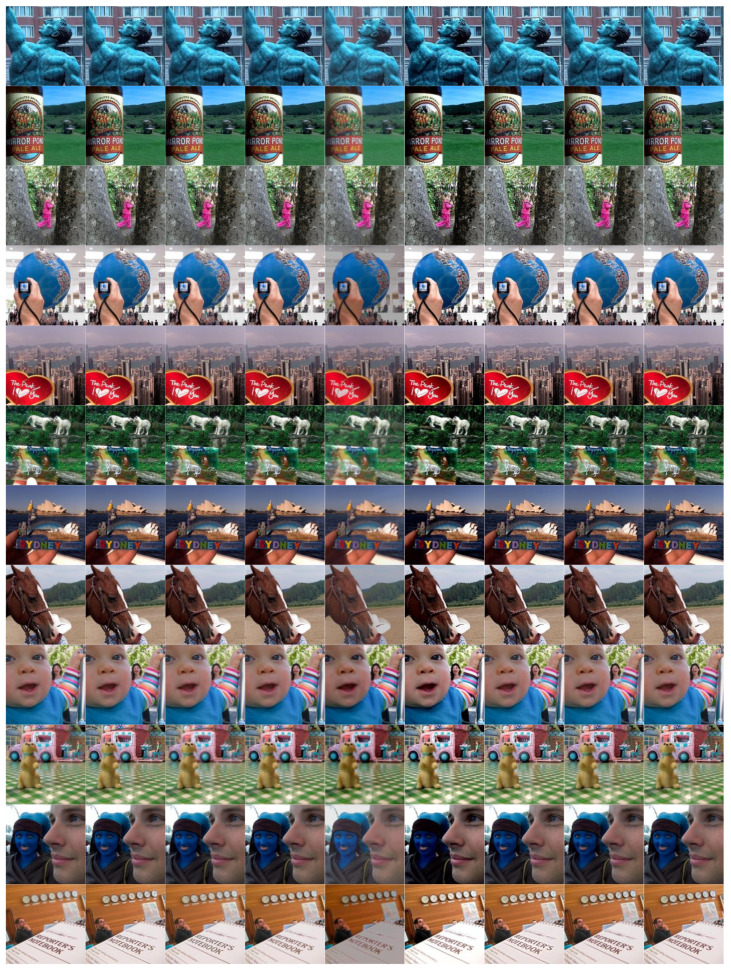

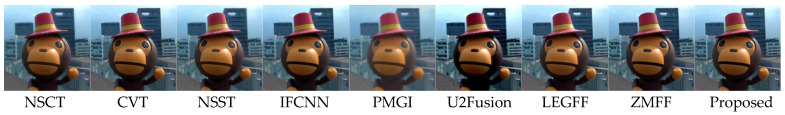
Fusion results on other images in Figure 2.

**Figure 8:**
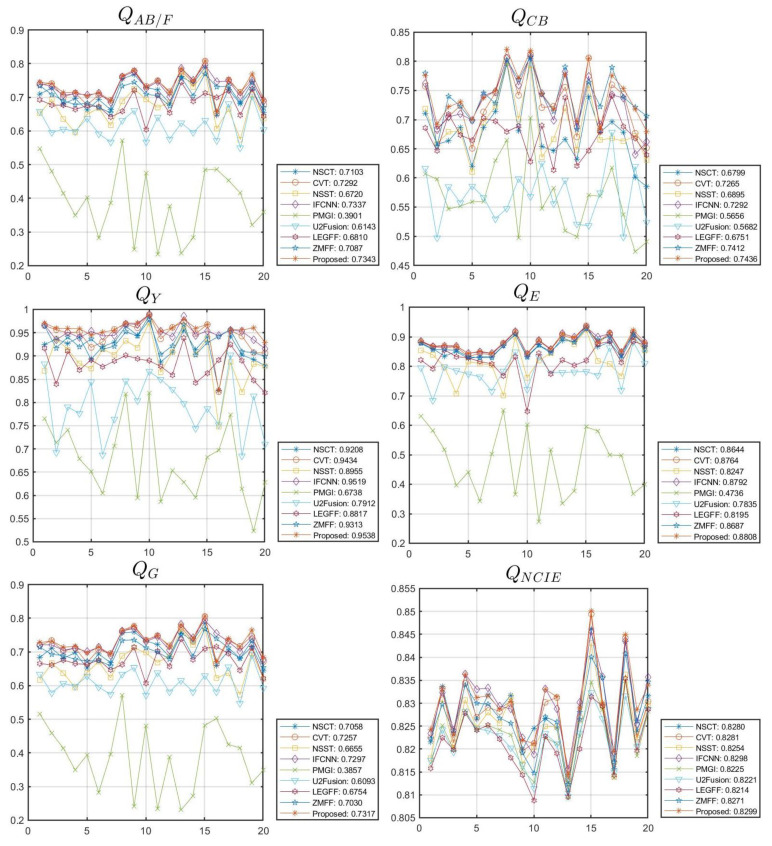

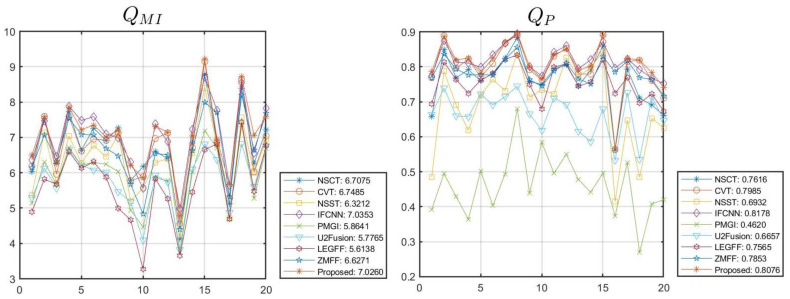
Line chart of metrics data with different methods in Figure 2.

In this section, 20 pairs of multi-focus images from the Lytro dataset [59] (Figure 2) are selected to experiment with the subjective and objective evaluation metrics to demonstrate the effectiveness of the proposed multi-focus image fusion algorithm. Compared with the latest published algorithms, we can highlight the advantages of our image fusion algorithm. The eight state-of-the-art image fusion methods are selected for comparison, and the methods are nonsubsampled contourlet transform and fuzzy-adaptive reduced pulse-coupled neural network (NSCT) [29], image fusion using the curvelet transform (CVT) [57], image fusion with parameter-adaptive pulse-coupled neural network in nonsubsampled shearlet transform domain (NSST) [36], image fusion framework based on convolutional neural network (IFCNN) [53], fast unified image fusion network based on the proportional maintenance of gradient and intensity (PMGI) [54], unified unsupervised image fusion network (U2Fusion) [55], local extreme map guided multi-modal image fusion (LEGFF) [52], and zero-shot multi-focus image fusion (ZMFF) [60]. A single image fusion evaluation index cannot fully reflect the image quality, and multiple evaluation indexes can be used together to more objectively analyze the data and image information. The eight metrics are used as the objective evaluation, and the metrics are the edge-based similarity measurement $Q_{AB/F}$ [61], the human perception inspired metric $Q_{CB}$ [62], the structural similarity-based metric $Q_{Y}$ introduced by Yang et al. [62], the structural similarity-based metric $Q_{E}$ [62], the gradient-based metric $Q_{G}$ [62], the nonlinear correlation information entropy $Q_{NCIE}$ [62], the mutual information $Q_{MI}$ [61], and the phase congruency-based metric $Q_{P}$ [62]. Figure 3, Figure 4, Figure 5, Figure 6 and Figure 7 show the corresponding fusion results, and Figure 8 and Table 1, Table 2, Table 3, Table 4 and Table 5 show the corresponding metrics data. In our method, the decomposition levels of the shearlet is 4, and the direction numbers are [10, 10, 18, 18]. The dictionary size is set to 256, and the iteration number of K-SVD is fixed to 180. The patch size is 6 × 6, the step length is set to 1, and the error tolerance ε is set to 0.1.

Figure 3 shows the fused images of different methods on the first pair of images in Figure 2, and Table 1 shows the corresponding metrics data. The fused images generated by the NSCT, CVT, and NSST algorithms are blurred in some areas. The PMGI method generates a dark image, and it is distorted and blurred. The IFCNN, U2Fusion, LEGFF, and ZMFF methods generate higher brightness. Compared with the other fusion methods, our method has the best fusion result, and more complementary image information is retained. The enlarged area in the images allows observing some details in the fused images. From Table 1, we can see that the metrics date of $Q_{AB/F}$, $Q_{Y}$, $Q_{E}$, $Q_{G}$, $Q_{NCIE}$, $Q_{MI}$, and $Q_{P}$ generated by our method are the best, and the corresponding values are 0.7446, 0.9708, 0.8868, 0.7273, 0.8243, 6.5008, and 0.7860, respectively. The ZMFF method generates the best value of $Q_{CB}$ with 0.7802, and our method, which achieves the value 0.7760, is ranked second.

Figure 4 shows the fused images of different methods on the second pair of images in Figure 2, and Table 2 depicts the corresponding metrics data. The fused images generated by the NSCT, CVT, IFCNN, LEGFF, and ZMFF algorithms produce a considerable fusion effect, and the images are similar. The NSST algorithm produces clearer close-range information, while the distant information, such as the outline of the mountain, is relatively fuzzy. The PMGI algorithm produces a fuzzy fusion image, which does not achieve the effect of information complementarity, and the definition is obviously low, so it is difficult to observe the details in the image. The U2Fusion method improves the brightness of some areas of the image, such as the man’s face area, but the head, mouth, and neck areas of the man are obviously dark, so it is impossible to observe these parts of the information. Compared with the other fusion algorithms, our algorithm obtained clear close and distant information, achieved the effect of information complementarity, and maintained the image details well, and the result is easy to observe in the image. From Table 2, we can see that the metrics date of $Q_{CB}$, $Q_{Y}$, and $Q_{E}$ computed by our method are the best, with the corresponding values 0.6924, 0.9593, and 0.8684, respectively.

Figure 5 shows the fused images of different methods on the third pair of images in Figure 2, and Table 3 shows the corresponding metrics data. The fused images generated by the NSCT and NSST algorithms are blurred in the girl’s face area. The CVT, IFCNN, LEGFF, and ZMFF methods generate all-focus images. The PMGI approach generates a distorted and blurred fusion image, making it impossible to obtain details in the images. Some areas in the fused image acquired by the U2Fusion method are very dark, such as the collar of the boys and girls, the tongue and hair of the boys, and the leaves. Our algorithm obtains a full-focus image, and the details of the source images are preserved well. From Table 3, we can see that the metrics date of $Q_{AB/F}$, $Q_{Y}$, $Q_{E}$, $Q_{G}$, and $Q_{P}$ generated by our method are the best, with the corresponding values 0.7134, 0.9589, 0.8710, 0.7139, and 0.8194, respectively.

Figure 6 shows the fused images of different methods on the third pair of images in Figure 2, and Table 4 shows the corresponding metrics data. The fused images generated by the NSCT, CVT, IFCNN, LEGFF, and ZMFF algorithms produce basic full-focus images. The NSST method has a blurred image, such as the contour information of the woman in the distance. The PMGI method produces a completely blurred effect, and it is dark. The U2Fusion method makes some areas too bright and some areas too dark, and does not achieve an effect of moderate brightness. Our method produces a clear full-focus image, and the information complementation achieves an optimal effect. From Table 4, we can see that the metrics date of $Q_{AB/F}$, $Q_{CB}$, $Q_{Y}$, $Q_{E}$, $Q_{G}$, and $Q_{P}$ generated by our method are the best, with the corresponding values 0.7148, 0.7301, 0.9584, 0.8691, 0.7162, and 0.8249, respectively.

Figure 7 shows the fused results of different methods on other images in Figure 2, and we can compare the fusion effect of different algorithms. Figure 8 shows the line chart of the metrics data with different methods in Figure 2, and we can observe the fluctuation of corresponding index values obtained by the different algorithms on the 20 groups of multi-focus images. The average metrics data of the different methods in Figure 8 are shown in Table 5, and from this table, we can notice that the metrics data $Q_{AB/F}$,$Q_{CB}$, $Q_{Y}$, $Q_{E}$, $Q_{G}$, and $Q_{NCIE}$ generated by the proposed method are the best. The values of $Q_{MI}$ and $Q_{P}$ generated by the IFCNN method are the best; however, the two corresponding index values $Q_{MI}$ and $Q_{P}$ obtained by our algorithm still rank second among all the algorithms and have obvious advantages. Through qualitative and quantitative evaluation and analysis, our algorithm achieves the best multi-focus image fusion effect.

## 5. Conclusions

In order to generate a clear full-focus image, a novel multi-focus image fusion method based on sparse representation and local energy in shearlet domain is introduced. The shearlet transform is utilized to decompose the source images into low- and high-frequency sub-bands; the sparse representation based fusion rule is used to fuse the low-frequency sub-band, and local energy based fusion rule is used to fuse the high-frequency sub-bands. Twenty groups of multi-focus images are tested, and the effectiveness of the algorithm proposed in this paper is verified through qualitative and quantitative evaluation and analysis. The average metrics data $Q_{AB/F}$, $Q_{CB}$, $Q_{Y}$, $Q_{E}$, $Q_{G}$, and $Q_{NCIE}$ computed by our method are the best, and the corresponding values are 0.7343, 0.7436, 0.9538, 0.8808, 0.7317, and 0.8299, respectively; the values of $Q_{MI}$ and $Q_{P}$ also generate relatively advanced data. In the future work, we will extend this algorithm to multi-exposure image fusion and other multi-modal image fusion.

## Figures and Tables

**Table 1:** Objective evaluation of methods in Figure 3.

	*Q_AB/F_*	*Q_CB_*	*Q_Y_*	*Q_E_*	*Q_G_*	*Q_NCIE_*	*Q_MI_*	*Q_P_*
NSCT	0.7092	0.7108	0.9248	0.8798	0.6831	0.8216	6.0272	0.6575
CVT	0.7373	0.7576	0.9652	0.8825	0.7191	0.8225	6.1919	0.7694
NSST	0.6526	0.7195	0.8680	0.8543	0.6167	0.8180	5.3646	0.4842
IFCNN	0.7412	0.7622	0.9666	0.8848	0.7207	0.8234	6.3637	0.7727
PMGI	0.5466	0.6070	0.7656	0.6316	0.5156	0.8169	5.1347	0.3925
U2Fusion	0.6575	0.6164	0.8832	0.7952	0.6338	0.8176	5.2894	0.6640
LEGFF	0.6923	0.6857	0.9164	0.8205	0.6658	0.8158	4.8919	0.6937
ZMFF	0.7342	**0.7802**	0.9644	0.8779	0.7134	0.8222	6.1505	0.7673
Proposed	**0.7446**	0.7760	**0.9708**	**0.8868**	**0.7273**	**0.8243**	**6.5008**	**0.7860**

**Table 2:** Objective evaluation of methods in Figure 4.

	*Q_AB/F_*	*Q_CB_*	*Q_Y_*	*Q_E_*	*Q_G_*	*Q_NCIE_*	*Q_MI_*	*Q_P_*
NSCT	0.7276	0.6578	0.9363	0.8598	0.7110	**0.8337**	7.5353	0.8483
CVT	**0.7411**	0.6838	0.9561	0.8661	**0.7332**	0.8333	**7.5911**	**0.8879**
NSST	0.6934	0.6588	0.9376	0.8391	0.6667	0.8308	7.1464	0.7872
IFCNN	0.7315	0.6825	0.9349	0.8663	0.7205	0.8324	7.4651	0.8744
PMGI	0.4798	0.5977	0.7132	0.5816	0.4592	0.8251	6.3071	0.4944
U2Fusion	0.5951	0.4969	0.6918	0.6838	0.5786	0.8242	6.1325	0.7393
LEGFF	0.6770	0.6466	0.8394	0.7920	0.6603	0.8225	5.8173	0.8132
ZMFF	0.7085	0.6544	0.9171	0.8568	0.6927	0.8297	7.0711	0.8365
Proposed	0.7404	**0.6924**	**0.9593**	**0.8684**	0.7326	0.8332	7.5773	0.8860

**Table 3:** Objective evaluation of methods in Figure 5.

	*Q_AB/F_*	*Q_CB_*	*Q_Y_*	*Q_E_*	*Q_G_*	*Q_NCIE_*	*Q_MI_*	*Q_P_*
NSCT	0.6800	0.6636	0.9272	0.8345	0.6807	0.8239	6.2537	0.7682
CVT	0.7008	0.7053	0.9498	0.8626	0.7020	0.8213	5.9563	0.7959
NSST	0.6359	0.6789	0.9138	0.7973	0.6372	0.8201	5.7004	0.6903
IFCNN	0.7060	0.7047	0.9509	0.8676	0.7047	**0.8241**	**6.4525**	0.8134
PMGI	0.4143	0.5467	0.7409	0.5178	0.4139	0.8198	5.6679	0.4286
U2Fusion	0.6059	0.5849	0.7903	0.7988	0.6064	0.8193	5.5648	0.6591
LEGFF	0.6764	0.7090	0.9104	0.8557	0.6757	0.8198	5.6745	0.7630
ZMFF	0.6898	**0.7406**	0.9408	0.8563	0.6890	0.8234	6.3327	0.7934
Proposed	**0.7134**	0.7222	**0.9589**	**0.8710**	**0.7139**	0.8231	6.2696	**0.8194**

**Table 4:** Objective evaluation of methods in Figure 6.

	*Q_AB/F_*	*Q_CB_*	*Q_Y_*	*Q_E_*	*Q_G_*	*Q_NCIE_*	*Q_MI_*	*Q_P_*
NSCT	0.6961	0.6866	0.9407	0.8496	0.6975	0.8363	7.7208	0.7916
CVT	0.7125	0.7240	0.9515	0.8656	0.7114	0.8343	7.5880	0.8219
NSST	0.5955	0.6809	0.8837	0.7067	0.5944	0.8308	7.0354	0.6179
IFCNN	0.7103	0.7098	0.9399	0.8679	0.7112	**0.8364**	**7.8860**	0.8101
PMGI	0.3491	0.5517	0.6784	0.3959	0.3491	0.8285	6.7140	0.3640
U2Fusion	0.5988	0.5576	0.7763	0.7853	0.5985	0.8282	6.6513	0.6573
LEGFF	0.6639	0.6739	0.8700	0.8327	0.6649	0.8279	6.5996	0.7240
ZMFF	0.6780	0.7229	0.9196	0.8539	0.6774	0.8340	7.5359	0.7762
Proposed	**0.7148**	**0.7301**	**0.9584**	**0.8691**	**0.7162**	0.8363	7.8484	**0.8249**

**Table 5:** Average metrics data of different methods in Figure 8.

	*Q_AB/F_*	*Q_CB_*	*Q_Y_*	*Q_E_*	*Q_G_*	*Q_NCIE_*	*Q_MI_*	*Q_P_*
NSCT	0.7103	0.6799	0.9208	0.8644	0.7058	0.8280	6.7075	0.7616
CVT	0.7292	0.7265	0.9434	0.8764	0.7257	0.8281	6.7485	0.7985
NSST	0.6720	0.6895	0.8955	0.8247	0.6655	0.8254	6.3212	0.6932
IFCNN	0.7337	0.7292	0.9519	0.8792	0.7297	0.8298	**7.0353**	**0.8178**
PMGI	0.3901	0.5656	0.6738	0.4736	0.3857	0.8225	5.8641	0.4620
U2Fusion	0.6143	0.5682	0.7912	0.7835	0.6093	0.8221	5.7765	0.6657
LEGFF	0.6810	0.6751	0.8817	0.8195	0.6754	0.8214	5.6138	0.7565
ZMFF	0.7087	0.7412	0.9313	0.8687	0.7030	0.8271	6.6271	0.7853
Proposed	**0.7343**	**0.7436**	**0.9538**	**0.8808**	**0.7317**	**0.8299**	7.0260	0.8076

## Data Availability

Not applicable.
